# Identifying Stable Reference Genes for qRT-PCR Normalisation in Gene Expression Studies of Narrow-Leafed Lupin (*Lupinus angustifolius* L.)

**DOI:** 10.1371/journal.pone.0148300

**Published:** 2016-02-12

**Authors:** Candy M. Taylor, Ricarda Jost, William Erskine, Matthew N. Nelson

**Affiliations:** 1 School of Plant Biology, The University of Western Australia, Crawley, Western Australia, Australia; 2 Department of Animal, Plant and Soil Sciences, ARC Centre of Excellence in Plant Energy Biology, School of Life Sciences, La Trobe University, Bundoora, Victoria, Australia; 3 Centre for Plant Genetics and Breeding, School of Plant Biology, The University of Western Australia, Crawley, Western Australia, Australia; 4 The UWA Institute of Agriculture, The University of Western Australia, Crawley, Western Australia, Australia; Murdoch University, AUSTRALIA

## Abstract

Quantitative Reverse Transcription PCR (qRT-PCR) is currently one of the most popular, high-throughput and sensitive technologies available for quantifying gene expression. Its accurate application depends heavily upon normalisation of gene-of-interest data with reference genes that are uniformly expressed under experimental conditions. The aim of this study was to provide the first validation of reference genes for *Lupinus angustifolius* (narrow-leafed lupin, a significant grain legume crop) using a selection of seven genes previously trialed as reference genes for the model legume, *Medicago truncatula*. In a preliminary evaluation, the seven candidate reference genes were assessed on the basis of primer specificity for their respective targeted region, PCR amplification efficiency, and ability to discriminate between cDNA and gDNA. Following this assessment, expression of the three most promising candidates [*Ubiquitin C* (*UBC*), *Helicase* (*HEL*), and *Polypyrimidine tract-binding protein* (*PTB*)] was evaluated using the NormFinder and RefFinder statistical algorithms in two narrow-leafed lupin lines, both with and without vernalisation treatment, and across seven organ types (cotyledons, stem, leaves, shoot apical meristem, flowers, pods and roots) encompassing three developmental stages. *UBC* was consistently identified as the most stable candidate and has sufficiently uniform expression that it may be used as a sole reference gene under the experimental conditions tested here. However, as organ type and developmental stage were associated with greater variability in relative expression, it is recommended using *UBC* and *HEL* as a pair to achieve optimal normalisation. These results highlight the importance of rigorously assessing candidate reference genes for each species across a diverse range of organs and developmental stages. With emerging technologies, such as RNAseq, and the completion of valuable transcriptome data sets, it is possible that other potentially more suitable reference genes will be identified for this species in future.

## Introduction

Transcriptome studies, including gene expression analyses, have become increasingly important for uncovering regulatory patterns in plant physiology, development, and metabolic responses to biotic and abiotic stresses [1,2]. Although several effective methods for quantifying gene expression currently exist, one of the most widely used technologies to date is quantitative Reverse Transcription Polymerase Chain Reaction (qRT-PCR) [3]. Among the advantages of this technique are its high-throughput capacity, high sensitivity and specificity, and broad dynamic range [2,4].

Specifically, qRT-PCR allows for the real-time detection and simultaneous quantification of transcript-derived complementary DNA (cDNA) products at the completion of each PCR cycle [5]. The quantification of cDNA products is achieved by the detection and measurement of a fluorescent signal. Most commonly, these signals are generated by DNA binding dyes (e.g. SYBR® Green), which bind to non-specific double stranded DNA, or DNA target-specific fluorescent reporter probes (e.g. TaqMan® probes) [6]. Once the level of fluorescence surpasses an arbitrarily assigned threshold, each sample is assigned a Threshold Cycle (C_T_) value [7]. As the rate at which the fluorescent signal increases during the exponential phase is directly dependent on the number of target cDNA copies present, the C_T_ value of each sample is inversely related to the initial amount of target cDNA at the beginning of the analysis [7,8]. Thus, qRT-PCR enables comparisons of relative gene expression.

The ability to produce reliable and accurate data through qRT-PCR is largely dependent on the use of suitable reference genes [4]. The inclusion of reference genes enables normalisation of gene-of-interest C_T_ values, effectively accounting for errors that may otherwise influence the determined level of gene expression within a sample. Such errors include variability in the initial volume or concentration of cDNA, RNA recovery or integrity, or efficiency of either cDNA synthesis or DNA polymerase enzymes [1].

There are a number of prerequisites for an effective reference gene [9]. The most important of these is that valid reference genes must exhibit stable and consistent levels of expression among various tissue/organ types and experimental conditions [10]. Previously, the most commonly used and endorsed reference genes were so-called housekeeping genes; *i*.*e*., genes responsible for the production of proteins directly involved in basic cellular functions and which consequently have consistent, uniform expression among various cell types and environmental conditions [1]. However, in recent years, the number of studies reporting variable expression of housekeeping genes has increased [11,12]. For example, genes encoding *β-actin* (*e*.*g*. [13–15]) and *glyceraldehyde 3-phosphate dehydrogenase* (*GAPDH*) (*e*.*g*., [16–18]) are known to be problematic for normalisation under certain experimental conditions. It is therefore not surprising that researchers are urged to validate candidate reference genes for their species-of-interest and the specific experimental conditions to be used [1,2].

Narrow-leafed lupin (*Lupinus angustifolius* L.) is a winter-annual legume and one of four domesticated lupin species collectively grown over 950,000 hectares of land globally in 2013 [19,20]. Traditionally, most lupin grain is grown as high-protein livestock feed. However, it is also an ancient pulse crop and considered as a potential human health food to counter obesity and diabetes due to its beneficially low glycaemic index, high fibre and protein content, as well as its ability to reduce insulin resistance [21,22]. Narrow-leafed lupin is a particularly important crop in Australia, the world’s leading producer of lupin grain since the mid 1980’s [21]. Narrow-leafed lupin’s success stems from its adaptation to sandy, acidic soils, which are highly prevalent within the Western Australian ‘grain belt’ [23]. Additionally, this species is a desirable component of crop rotations due its capacity to improve soil fertility via symbiotic fixation of atmospheric nitrogen and mobilization of soil-bound phosphorus [24,25].

As a recently domesticated species [21], much has still to be learned about narrow-leafed lupin in all fields of plant biology. Given the limited genetic diversity of this crop and its vulnerability to predicted climate change within the Australian landscape [26], understanding the roles and regulation of individual genes will be a particularly important objective for researchers. As a result, transcriptome and gene expression studies of narrow-leafed lupin (such as that of Przysiecka *et al*. [27]) are likely to increase in number and be of great future value.

The purpose of this study is to provide an independent recommendation of suitable reference genes for normalisation in qRT-PCR analyses of narrow-leafed lupin. Here, we have evaluated seven housekeeping genes previously trialed as candidate reference genes for the model legume, *Medicago truncatula*, by Kakar *et al*. [28]. In that study, Kakar *et al*. compared 13 potential reference genes for stable expression across six organ types at up to three stages of maturity in vernalised *M*. *truncatula* plants. Our analysis incorporated a preliminary testing of the candidate reference genes for primer specificity, PCR amplification efficiency, and primer ability to discriminate between genomic DNA (gDNA) and cDNA of narrow-leafed lupin. The three most promising candidates were then evaluated using the NormFinder [29] and RefFinder [30] algorithms for stable gene expression with a greater number of factors, including: firstly, testing for consistent expression in two lines of narrow-leafed lupin (a representative wild and representative domestic line); secondly, stability within and across seven organ types; thirdly, stability of expression over time (via collection of organs at three developmental stages); and lastly, consistent expression with and without vernalisation (*i*.*e*., a prolonged period of exposure to cold winter temperatures, which enables floral competency in warm spring conditions [31]). Based on these results, we present our recommendations for suitable reference genes for qRT-PCR studies of narrow-leafed lupin.

## Methods

### Plant growth and harvest

Four recombinant inbred lines (RILs) sampled randomly from an F_8_ RIL population developed by the Department of Agriculture and Food Western Australia (Perth, Australia) and their parental lines, 83A:476 (a domesticated breeding line) and P27255 (a wild Moroccan accession), were grown for the purposes of this study [32]. All seeds were initially scarified and imbibed in purified (Milli-Q)-water for a period of 6 hours and then divided into vernalised and non-vernalised treatments. Approximately one half of the seeds of each parent were then directly sown into pots (non-vernalised treatment). A vernalisation treatment involving 21 days’ incubation at 4°C in a darkened, temperature-controlled room was imposed on the remaining parental seeds and all RIL seeds. Following this treatment, the vernalised seedlings were transferred to potting containers. All pots were housed within a phytotron located at The University of Western Australia (Crawley, Australia; 31°59' S, 115°49' E). The phytotron was maintained within a diurnal temperature range of 14±0.5°C (night) and 18±0.5°C (day), and was exposed to the natural photoperiod (ranging from approximately 10 h—11.75 h) during April to September 2014. The plants were watered once daily and fertilized once per fortnight using 2 g of Osmocote® Water Soluble General Purpose Fertiliser (Scotts Pty Ltd).

As summarised in Table 1, seven organ types were harvested from the RILs and their parental lines. Harvest of the four RILs encompassed a range of plant maturities. Two biological replicates each were sampled of the following organs: fully emerged leaves; stem; shoot apical meristems (SAMs); flowers; and pods. As described by Dracup & Kirby [33], a fully emerged leaf was defined as having leaflets that had begun to unfold and which no longer fully contacted each other. Organs were harvested from the parental line plants at three distinct developmental stages: firstly, the vegetative stage, in which seedlings had developed six fully emerged leaves; secondly, the early reproductive stage, which occurred 14 days from the development of six fully emerged leaves; and lastly, the late reproductive stage, in which plants had flowered and developed one or more pods of approximately 2.5 cm in length. Three biological replicates were harvested per parental line for each of the seven organ types sampled. Additionally, the SAM and leaves were harvested at two and three developmental stages, respectively. All samples were immediately frozen in liquid nitrogen and stored at -80°C prior to RNA extraction.

**Table 1:** A summary of seven organ types harvested at selective stages of plant development from six lines of narrow-leafed lupin, including four randomly selected F_8_ recombinant inbred lines (RILs) and their parental lines, 83A:476 and P27255.

Organ	Description	Parental lines			RILs
		Vegetative^a^	Early reproductive^b^	Late reproductive^c^	Variable development stages^d^
Cotyledons	Both cotyledons	X			
Leaves	The four uppermost fully expanded leaves (*i*.*e*., leaves with fully unfolded leaflets)	X	X	X	X
Stem	Approximately 3–4 cm of stem, including one or more nodal regions		X		X
Shoot Apical Meristem (SAM)	c. 1 cm of most distal stem, including the shoot apex		X	X	X
Flowers	Three to four hooded (*i*.*e*., unopened) flowers from the primary inflorescence			X	X
Pods	One to two pods, approximately 2.5 cm in length, from the primary inflorescence			X	X
Roots	c. 1–2 cm of tap root with adjoining lateral roots		X		

^a^ The vegetative developmental stage was defined as being when plants had developed six leaves, each with fully unfolded leaflets that were no longer contacting each other.

^b^ The early reproductive stage was defined as occurring fourteen days following the development of six leaves with fully unfolded leaflets.

^c^ The late reproductive stage was defined as when plants had flowered and developed one or more pods of 2.5 cm in length.

^d^ Plants were of various growth stages, including pre- and post-flowering.

### RNA extraction and cDNA synthesis

Total RNA was isolated from organ samples using the Spectrum^TM^ Plant Total RNA Kit (Sigma Aldrich Inc.) with three modifications to the manufacturer’s instructions. Firstly, due to the small mass of the SAM and flower samples, as little as 30–100 mg of frozen, ground powder was used for RNA isolation from these organ types. Secondly, to eliminate inhibitors of cDNA synthesis and/or cDNA amplification, the first column wash was repeated for all samples. Lastly, 70 μL of elution solution was applied to the RNA binding column to encourage more consistent RNA recovery. Concentrations of RNA were determined using the NanoDrop 1000 Spectrophotometer (Thermo Fisher Scientific Inc.) and Qubit® 2.0 Fluorometer (Qubit® RNA HS Assay Kit; Invitrogen^TM^). There was a 0.80 positive correlation between the two independent estimates of RNA concentration, with NanoDrop estimates being on average 33% higher than Qubit estimates (data not presented). As the RNA yield of the non-vernalised P27255 cotyledons was initially low, RNA was precipitated to increase final concentration.

First-strand cDNA synthesis was then conducted using the Tetro cDNA Synthesis Kit (Bioline Pty Ltd.), as per the manufacturer’s instructions. The 20 μL reactions contained approximately 2 μg of RNA and equal amounts of oligo (dT) and random hexamer primers (60 μM each). All cDNA samples were then diluted 10-fold before being used in qRT-PCR analyses.

### Primer design

Several housekeeping genes previously tested by Kakar *et al*. [28] as candidate reference genes for the model legume, *M*. *truncatula*, were trialed as candidates for narrow-leafed lupin in this study. The selected genes included: *β-tubulin* (*TUB*), *Helicase* (*HEL*), *Protodermal factor 2* (*PDF2*), *Pentatricopeptide repeat protein* (*PPR*), *Polypyrimidine tract-binding protein* (*PTB*), *Ubiquitin C* (*UBC*), and *Ubiquitin-protein ligase 7* (*UPL7*). The *M*. *truncatula* tentative consensus (TC) sequence for each gene was retrieved from: *compbio*.*dfci*.*harvard*.*edu/tgi/cgi-bin/tgi/gimain*.*pl*?*gudb = medicago/*. Homologous sequences for narrow-leafed lupin were then located within the unigene assembly developed from transcriptome sequencing of cv. Tanjil [34] using BLASTn search in Geneious 7.0 (Biomatters Ltd.) (S1 Table). Strict parameters were used to identify the BLASTn hit with greatest homology and included: the full/partial transcript being >350 bp in length; an E-score <1e-20; and greatest homology to *M*. *truncatula* according to the Bit score and pairwise identities for the transcript and deduced amino acid sequences. The *M*. *truncatula* primer sequences designed by Kakar *et al*. were then aligned to their respective homologous sequences in narrow-leafed lupin. Any mismatching nucleotides within primer sequences were replaced to complement the narrow-leafed lupin transcript with highest sequence homology. Additionally, the primers were lengthened or shifted slightly where they did not meet the criteria for real-time primer design, including: a melting temperature of approximately 60°C; an ideal GC content of 40–60%; length of approximately 20–35 bp; limited self- and hetero-dimer formation; and amplicon sizes of 100 to 150 bp. Sequences for the narrow-leafed lupin qRT-PCR primers are presented in Table 2.

**Table 2:** Quantitative Reverse Transcription PCR (qRT-PCR) primers designed for seven candidate reference genes in narrow-leafed lupin (*Lupinus angustifolius* L.).

Reference gene	*Medicago truncatula* TC ^a^	*Lupinus angustifolius* transcript code ^b^	Primer direction	*Lupinus angustifolius* primer sequence (5’ → 3’)	T_m_ (^o^C)	Product length (bp)
*TUB*	TC191797	comp613_c0_seq3	Forward	TTTGCACCCCTTACCTCCC	59.2	101
			Reverse	GCAGCACACATCATGTTTTTGG	59.5	
*HEL*	CB892427	comp11345_c0_seq1	Forward	TTGTACGAGGTCGGTGCTCT	60.9	127
			Reverse	ACAAGCAACCAAATATTGCACCATA	60.0	
*PDF2*	TC181279	comp7642_c0_seq1	Forward	TGTGTTGCTTCAACCATTGGAAA	59.8	109
			Reverse	ATCTTGTTCCCTCATCTGAGCA	59.2	
*PPR*	TC96273	comp39030_c0_seq1	Forward	GGAAGACTAGAGGCTGCACAAG	60.7	113
			Reverse	AACAAACCCTCTTTACAAAATCCGTT	60.1	
*PTB*	TC111751	comp12954_c0_seq1	Forward	TGGAAGCTGGAGATGTTAGCATT	60.1	113
			Reverse	CTGAATCAGAGCCTGGAAACCT	60.0	
*UBC*	AW686873	comp4884_c0_seq1	Forward	CTGACAGCCCACTGAATTGTGA	60.8	108
			Reverse	TCTTGGGCATAGCAGCAAGC	61.0	
*UPL7*	TC111218	comp15438_c0_seq1	Forward	CCAGTTGTTCTCGTGCTCCAT	60.6	104
			Reverse	TCCTCCGATTGTTGCCCAAA	59.9	

Primers designed by Kakar *et al*. [28] for *Medicago truncatula* (using gene sequences corresponding to the Tentative Consensus (TC) sequences^a^ provided) served as templates and were modified to complement narrow-leafed lupin transcripts^b^ with highest sequence homology obtained from the Tanjil unigene assembly [34]. Note that the *M*. *truncatula* TC sequences for *TUB* and *PDF2* were altered from those presented by Kakar *et al*. [28] when retrieving sequences from: *compbio.dfci.harvard.edu/tgi/cgi-bin/tgi/gimain.pl?gudb=medicago/*.

### Quantitative Reverse-Transcription PCR

Quantitative Reverse-Transcription PCR was performed and analysed using the Applied Biosystems® 7500 Fast Real-Time PCR System and accompanying Applied Biosystems® 7500 Software (version 2.0.6). Each qRT-PCR contained 25 ng of cDNA, 3 pmoles each of forward and reverse primers, and 5 μL of KiCqStart® SYBR® Green qPCR ReadyMix^TM^ and water added to give a total volume of 10uL. Two No-Reverse Transcription (No-RT) controls were loaded per 96-well plate to check for undesirable gDNA amplification. The PCR programming comprised an initial denaturation at 95°C for 30 seconds, followed by 40 cycles of denaturation at 95°C for three seconds and primer annealing and extension at 65°C for 30 seconds. Melt-curve analyses were then conducted before concluding the program with a 4°C hold.

### Assessment of primer specificity, efficiency, and discrimination

Preliminary evaluation of the candidate reference gene primers was made via assessment of primer specificity, efficiency and discrimination between F_8_ RIL cDNA and gDNA, as summarised in Table 3. Evaluation of primer specificity and efficiency was made using two technical replications of two biological replicates per organ type. Assessment of primer discrimination included two technical replicates of one biological replicate per organ type.

**Table 3:** A summary of the preliminary evaluation conducted for quantitative Reverse-Transcription PCR (qRT-PCR) primers of narrow-leafed lupin candidate reference genes.

Primer characteristic	Testing method	Description of method
Specificity	Melt curve-analyses	The presence of one melt-curve peak indicated amplification of a single product and the absence of significant primer-dimer formation.
	Agarose and polyacrylamide gel electrophoresis of PCR product	Primer specificity for targeted regions was indicated by the presence of a singular product ofappropriate size on a high resolution agarose gel (3% v/w) run at 180 V for 40 minutes. Absence of primer dimer formation was confirmed using a non-denaturing polyacrylamide gel (20% w/v) electrophoresis at 100V for 3 hours.
Efficiency	LinRegPCR^a^ efficiency and R^2^ determination	Efficiencies within the range of 1.8–2.0 were considered ideal for qRT-PCR. R^2^ values greater than 0.98 indicated efficiencies were reliably determined.
Discrimination between cDNA & gDNA	qRT-PCR of No-Reverse Transcription controls	C_T_ values of 40 (*i*.*e*., undetermined C_T_) in the No-Reverse Transcription controls (which contained 25 ng of RNA in place of cDNA) indicated the ability for primers to sufficiently discriminate between DNA species.

^a^ For information on LinRegPCR (version 11.0) [35], please refer to: http://download.gene-quantification.info/.

### Assessment of candidate reference gene expression level and stability

The three most promising candidate genes from the preliminary evaluation (Table 3) were further tested for stable gene expression in the parental lines under a number of factors. These factors included: lupin parental line; vernalisation treatment; organ type; and developmental stage at time of harvest. A total of 124 organ samples were assessed, with a minimum of nine biological replicates per organ type (S3 Table).

The average C_T_ values and standard deviation were calculated for each candidate reference gene as a means of determining the overall variability of gene expression across the four factors. Unbalanced ANOVAs calculated using GenStat (Release 16.2 –VSN International) were used to identify if the four factors were associated with significant differences in the relative expression of candidate genes. The NormFinder [29] (freely available at: http://moma.dk/normfinder-software) and RefFinder [30] (freely available at: http://fulxie.0fees.us/?type=reference) algorithms were then used to identify the most effective candidate reference gene for normalisation and rank the three candidate reference genes in descending order of stability. NormFinder was selected as a robust tool capable of assessing the candidate reference genes according to their inter- and intra-group variation between and within factors, respectively [1,13,29]. Meanwhile, RefFinder was chosen for its ability to integrate the four most popular and widely used statistical approaches (Comparative Delta-Ct [36], BestKeeper [37], NormFinder, and geNorm [38]) to complement the ranking assessments by NormFinder. Additionally, NormFinder was used to identify which two candidate reference genes formed the most reliable pair of internal controls for normalisation for each factor, and RefFinder was used to identify which candidates were most stable for each individual level within the four factors (organ type, developmental stage, vernalisation treatment and parental line). Three samples with outliers for only one of the three candidate reference genes were excluded from analysis in RefFinder.

## Results

### Assessment of primer efficiency and target specificity

**Fig 1:**
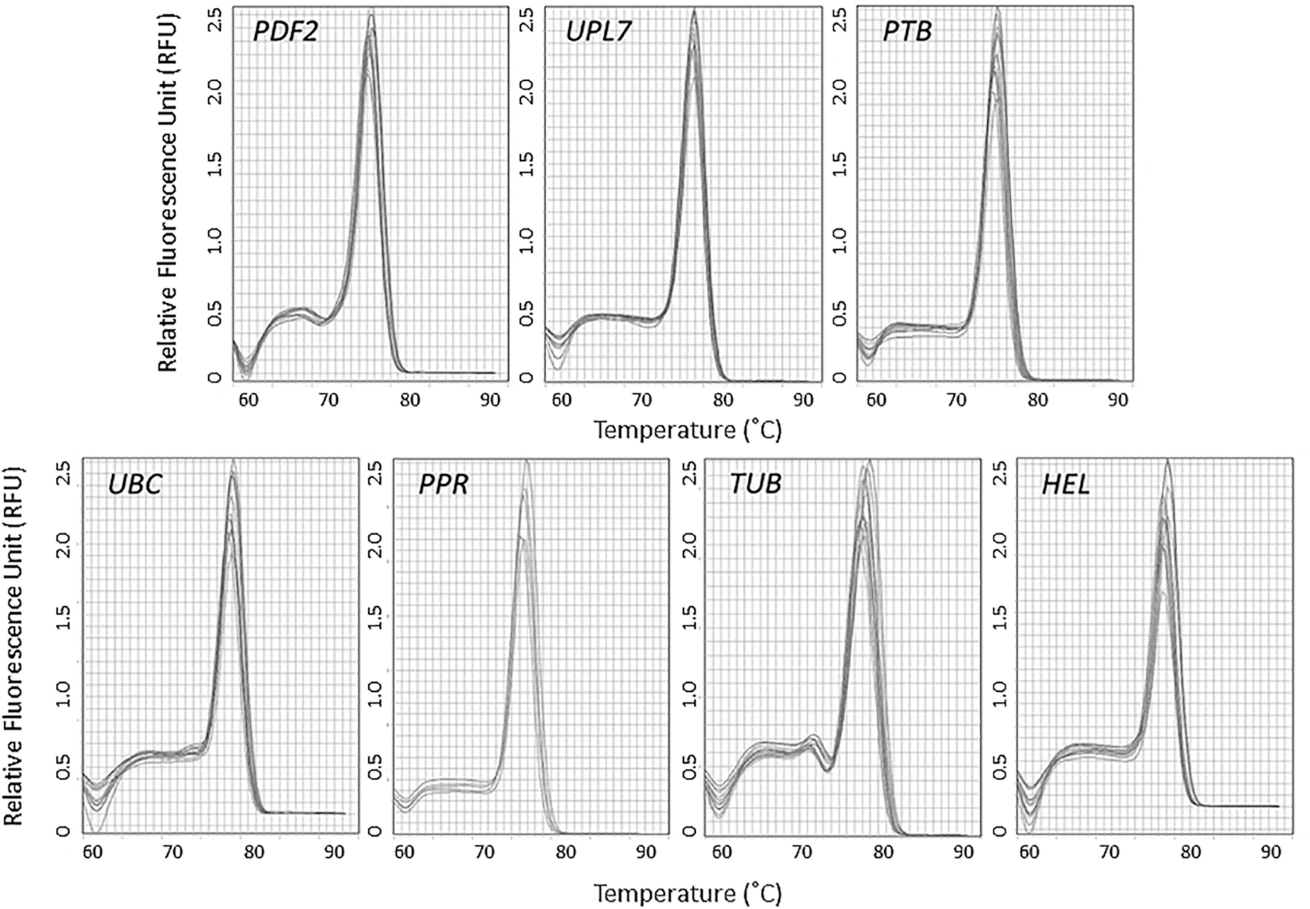
Melt curve analyses of *PDF2*, *UPL7*, *PTB*, *UBC*, *PPR*, *TUB* and *HEL* candidate reference genes for quantitative Reverse-Transcription PCR (qRT-PCR) of narrow-leafed lupin.

**Fig 2:**
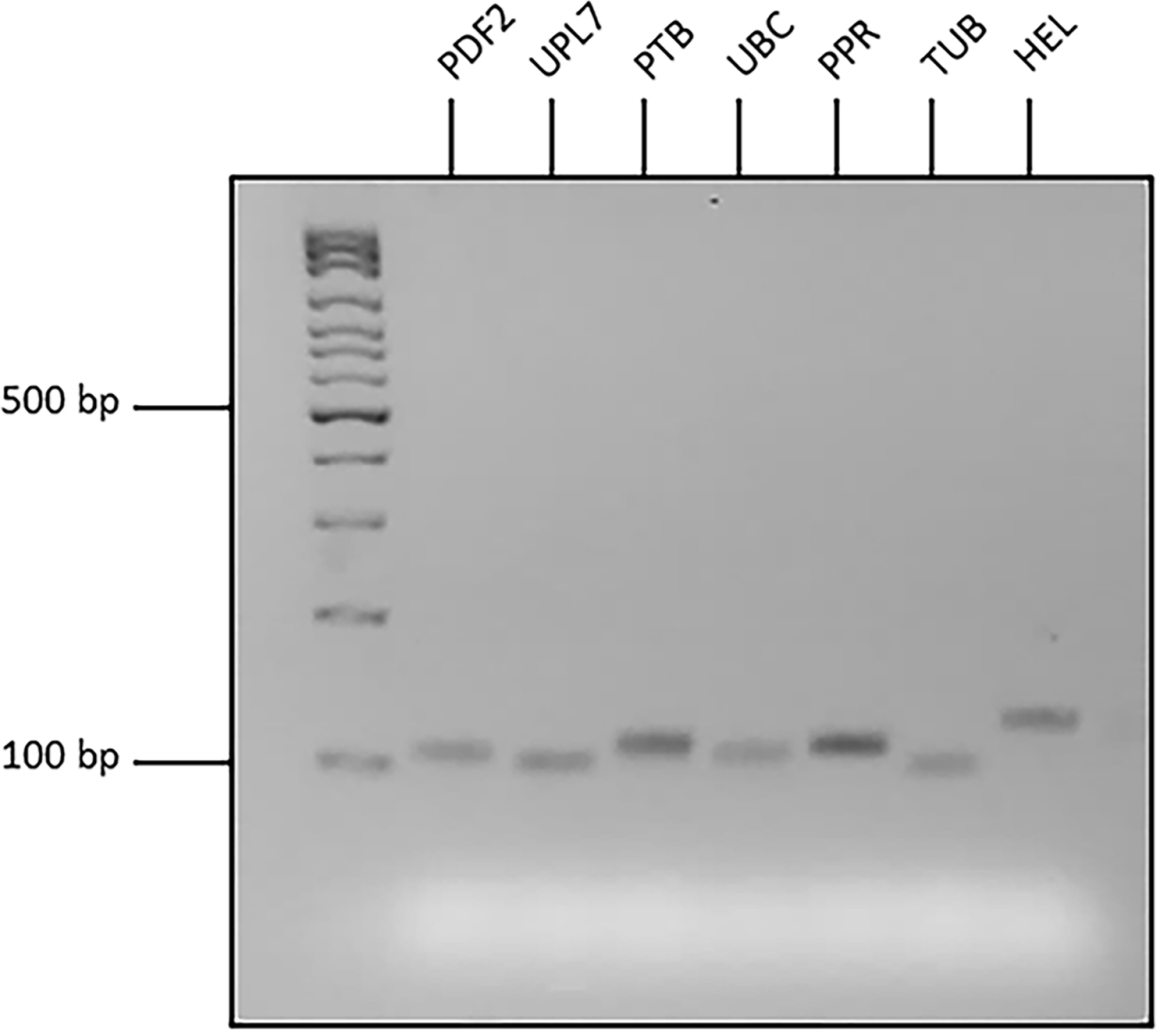
Gel electrophoresis of quantitative Reverse-Transcription PCR (qRT-PCR) products amplified using primers for seven narrow-leafed lupin candidate reference genes. The 3% (w/v) low-melt agarose, 1x TBE gel and was supplied with 180 V for 40 minutes. A 100 bp ladder (Ayxgen®) was used to determine approximate sizes of qRT-PCR products.

Initial assessment of seven candidate reference genes was conducted using organ samples from four RILs of narrow-leafed lupin. For each primer set, a single peak was observed in the melt curve analyses of both biological and technical replicates of each organ type (Fig 1). This indicated that unique products were amplified by all primer pairs during qRT-PCR. Agarose gel electrophoresis (Fig 2) confirmed that the single products amplified were of expected length (Table 1). A high resolution polyacrylamide gel revealed the absence of primer dimer formation for all candidate reference gene primers (data not presented).

The PCR efficiency of each of the seven primer pairs was to a very high standard, with all primer pairs reporting average efficiencies between 1.945 and 1.996 (Table 4). Additionally, the R^2^ values showed very little variation, with all reference genes reporting average values greater than 0.998. Thus, there was good reason to believe these estimated efficiencies were both sufficient for qRT-PCR and accurately determined.

**Table 4:** Average PCR efficiency and R^2^ values (± Standard Deviation) for seven candidate reference genes of narrow-leafed lupin.

Reference gene	PCR efficiency (± SD)	R^2^ (± SD)
*PDF2*	1.94 ± 0.15	0.9993 ± 0.0007
*UPL7*	1.98 ± 0.07	0.9996 ± 0.0004
*PTB*	1.96 ± 0.05	0.9999 ± 0.0001
*UBC*	1.99 ± 0.06	0.9998 ± 0.0002
*PPR*	1.99 ± 0.12	0.9990 ± 0.0003
*TUB*	1.97 ± 0.05	0.9997 ± 0.0003
*HEL*	1.94 ± 0.09	0.9998 ± 0.0002

Lastly, the seven primer sets were divided into three groups on the basis of their ability to discriminate between cDNA and gDNA. The first group of candidate reference genes, which did not amplify gDNA in the No-RT controls, included *PTB*, *UBC* and *HEL*. Primers belonging to the second group were able to amplify very small amounts of gDNA in most of the No-RT controls. This group included *PDF2* and *UPL7*, which reported average C_T_ values (and standard deviations) of 39.86 ± 0.30 and 37.54 ± 1.63 in No-RT controls, respectively. The final group of candidate reference genes included those with primers that displayed a complete inability to discriminate between cDNA and gDNA, and which achieved high amplification levels for both DNA species. As primer pairs targeting *PPR* and *TUB* achieved average C_T_ values of approximately 25 and 25.5 across an initial set of No-RT samples, respectively, both candidate reference genes fell into this latter group.

### Gene expression level and stability

Following the initial evaluation of our seven primer sets in the RILs, the following candidates were selected as the most promising reference genes for further testing: *PTB*, *UBC* and *HEL*. As all seven candidate reference genes performed exceptionally well in terms of specificity and efficiency, this selection was made on the basis of primers being able to discriminate unambiguously between gDNA and cDNA. Further evaluations incorporating more factors were then conducted to determine the relative level and stability of expression for *PTB*, *UBC* and *HEL*. The additional factors included: firstly, assessment of expression stability in the two parental lines of narrow-leafed lupin, P27255 and 83A:476, respectively; secondly, assessment of expression stability in vernalised and non-vernalised plants, with vernalised seeds subjected to a 4°C incubation for three weeks; and lastly, stable expression throughout the life-time of the plants, as determined by harvesting the seven organs at one or more of three developmental stages.

The expression of all three candidate reference genes was to a sufficiently high level. The most highly expressed candidate was *UBC*, with an overall average C_T_ value of 20.03 ± 1.76 (standard deviation). Both *PTB* and *HEL* were expressed approximately 8-fold less than *UBC*, with average C_T_ values and standard deviations of 23.02 ± 2.06 and 23.69 ± 1.62, respectively.

Relative expression of each candidate was clearly variable between organ types. The difference between minimum and maximum average C_T_ values across organ types for *PTB*, *UBC* and *HEL* was approximately 2.4, 2.0 and 2.1, respectively (Table 5). Such differences equate to approximately 4-fold differences in expression. Notably, the greatest expression differences occurred between the roots and reproductive organs, including the flowers and pods, whilst expression levels were more consistent between organs that were physically close to each other on the plant. For example, expression of all three candidate reference genes in flowers and pods was similar, as was the relative level of expression in vegetative organs, such as the SAM and stems or cotyledons and leaves. Not surprisingly, organ type was identified as the most significant factor to influence relative expression for all three candidate reference genes (p<0.001; S4 Table).

**Table 5:** Average C_T_ value with standard deviation for three candidate reference genes in different lupin organs.

Organ type	Mean C_T_ value ± standard deviation		
	*PTB*	*UBC*	*HEL*
Cotyledons	23.61 ± 0.94	20.92 ± 0.43	23.72 ± 0.87
Leaves	23.78 ± 1.70	20.45 ± 1.18	24.76 ± 1.00
Stem	22.54 ± 2.40	19.43 ± 2.17	22.85 ± 2.11
SAM	22.13 ± 1.83	20.02 ± 2.02	23.09 ± 1.44
Root	24.47 ± 1.72	20.95 ± 1.35	24.69 ± 1.60
Flower	22.42 ± 2.87	18.99 ± 1.10	22.67 ± 1.27
Pod	22.04 ± 1.57	19.46 ± 2.63	23.22 ± 2.26

The next most important factor to influence expression of all three candidate genes was parental line. Across all organ types, the average expression of *PTB*, *UBC* and *HEL* was 1.93- (p = 0.003), 1.76- (p = 0.003) and 1.62-fold (p = 0.001) greater in the domestic parent (83A:476) relative to the wild parent (P27255) (S4 Table). With respect to individual organ types, significant differences between the two parental lines occurred within the stem, roots, pods, leaves and SAM and typically involved either *PTB* and/or *UBC* (S5 Table). A strong interaction between parental line and developmental stage was also present within the leaves and SAM for all three genes (S6 Table), which suggested that parental line influenced temporal changes in expression.

The influence of vernalisation treatment and developmental stage on relative expression of the candidate reference genes was organ specific. Broadly, *HEL* was the only candidate gene to report significant differences among developmental stages (with respect to leaf and SAM organs only) and vernalisation treatments (when considered across all organ types) (S4 and S6 Tables). However, when analysing organs on an individual basis, vernalisation resulted in significant differences in the expression of *PTB* within the pods (p = 0.07), *UBC* within the leaves (p = 0.012) and roots (p = 0.039), and *HEL* within the stems (p = 0.049) (S5 Table). Additionally, developmental stage was a significant factor for *UBC* and *HEL* within the leaves (p = 0.013 each) and not SAM (S5 Table).

Using the NormFinder algorithm, our three candidate reference genes were ranked according to their overall level of stability across all samples with consideration to each factor (Table 6). Importantly, *UBC* was consistently identified as the most stable candidate reference gene, irrespective of the factor considered. *PTB* was in all cases identified as the third most stable candidate reference gene.

**Table 6:** Ranking of three candidate reference genes in descending order of expression stability, as calculated by NormFinder, with consideration to one of four factors: parental line, vernalisation treatment, organ type, and developmental stage.

Parental line		Vernalisation treatment		Organ type		Developmental stage	
*UBC*	0.058	*UBC*	0.057	*UBC*	0.133	*UBC*	0.105
*HEL*	0.109	*HEL*	0.108	*HEL*	0.177	*HEL*	0.105
*PTB*	0.432	*PTB*	0.275	*PTB*	0.320	*PTB*	0.316

Values presented are NormFinder Stability Values, in which a lower value indicates greater stability. Commonly reported Stability Values for the most stable, single reference gene in published data sets range between approximately 0.02 to 0.5 (*e.g.[1,2,9,10,18,39–42]*).

Similarly to the NormFinder algorithm, RefFinder determined *UBC* as the overall most stable gene across all data, closely followed by *HEL* and lastly by *PTB* (Table 7). Each of the four statistical approaches integrated within RefFinder also agreed with this ranking with the exception of BestKeeper, which identified *HEL*, *UBC* and *PTB* as the most stable genes listed in descending order. The use of RefFinder provided an interesting insight into candidate reference gene stability within levels of each factor. Across the fourteen levels tested, *UBC* was most frequently identified as the most stable candidate gene and *PTB* the least stable.

**Table 7:** Expression stability values and overall rankings for three narrow-leafed lupin candidate reference genes (*PTB*, *UBC* and *HEL*), as calculated by the RefFinder, which integrates the Comparative Delta Ct, BestKeeper, NormFinder and geNORM statistical programs. Stability values are calculated for each statistical program using unique algorithms, and are therefore not comparable between programs. Rankings of candidate reference genes are in descending order of expression stability.

Factor	Level	Program	Stability values			Ranking		
			*PTB*	*UBC*	*HEL*	1st	2nd	3rd
All data		RefFinder	3.000	1.189	1.414	UBC	HEL	PTB
		Comparative Delta Ct	1.580	1.428	1.435	UBC	HEL	PTB
		BestKeeper	1.548	1.340	1.295	HEL	UBC	PTB
		NormFinder	1.294	0.896	0.918	UBC	HEL	PTB
		geNORM	1.481	1.283	1.283	UBC/HEL	-	PTB
Organ type	Cotyledon	RefFinder	1.861	2.280	1.189	HEL	PTB	UBC
		Comparative Delta Ct	0.665	0.909	0.605	HEL	PTB	UBC
		BestKeeper	0.697	0.290	0.661	UBC	HEL	PTB
		NormFinder	0.417	0.875	0.180	HEL	PTB	UBC
		geNORM	0.360	0.360	0.726	HEL/PTB	-	UBC
	Flower	RefFinder	3.000	1.000	1.682	UBC	HEL	PTB
		Comparative Delta Ct	2.541	1.752	1.977	UBC	HEL	PTB
		BestKeeper	1.991	0.808	0.886	UBC	HEL	PTB
		NormFinder	2.409	0.594	1.361	UBC	HEL	PTB
		geNORM	2.090	1.188	1.188	UBC/HEL	-	PTB
	Leaf	RefFinder	3.000	1.189	1.414	UBC	HEL	PTB
		Comparative Delta Ct	1.214	1.049	1.065	UBC	HEL	PTB
		BestKeeper	1.093	0.846	0.610	HEL	UBC	PTB
		NormFinder	1.034	0.605	0.667	UBC	HEL	PTB
		geNORM	1.110	0.900	0.900	UBC/HEL	-	PTB
	Pod	RefFinder	1.000	3.000	1.682	PTB	HEL	UBC
		Comparative Delta Ct	1.148	1.516	1.246	PTB	HEL	UBC
		BestKeeper	1.074	1.665	1.291	PTB	HEL	UBC
		NormFinder	0.297	1.386	0.827	PTB	HEL	UBC
		geNORM	0.878	1.303	0.878	HEL/PTB	-	UBC
	Root	RefFinder	1.316	1.414	2.711	UBC	PTB	HEL
		Comparative Delta Ct	0.857	0.956	1.154	PTB	UBC	HEL
		BestKeeper	1.413	1.061	1.278	UBC	HEL	PTB
		NormFinder	0.329	0.666	1.060	PTB	UBC	HEL
		geNORM	0.659	0.659	0.989	UBC/PTB	-	HEL
	SAM	RefFinder	2.711	1.861	1.000	HEL	UBC	PTB
		Comparative Delta Ct	2.012	1.639	1.508	HEL	UBC	PTB
		BestKeeper	1.431	1.708	1.107	HEL	UBC	PTB
		NormFinder	1.849	1.082	0.342	HEL	UBC	PTB
		geNORM	1.720	1.135	1.135	UBC/HEL	-	PTB
	Stem	RefFinder	1.861	1.000	2.711	UBC	PTB	HEL
		Comparative Delta Ct	1.252	1.216	1.692	UBC	PTB	HEL
		BestKeeper	1.799	1.574	1.781	UBC	HEL	PTB
		NormFinder	0.651	0.422	1.601	UBC	PTB	HEL
		geNORM	0.775	0.775	1.386	UBC/PTB	-	HEL
Parental line	83A:476	RefFinder	3.000	1.000	1.682	UBC	HEL	PTB
		Comparative Delta Ct	1.653	1.342	1.423	UBC	HEL	PTB
		BestKeeper	1.452	1.205	1.306	UBC	HEL	PTB
		NormFinder	1.456	0.593	0.941	UBC	HEL	PTB
		geNORM	1.473	1.112	1.112	UBC/HEL	-	PTB
	P27255	RefFinder	1.861	2.711	1.000	HEL	PTB	UBC
		Comparative Delta Ct	1.505	1.511	1.433	HEL	PTB	UBC
		BestKeeper	1.550	1.497	1.269	HEL	UBC	PTB
		NormFinder	1.111	1.127	0.894	HEL	PTB	UBC
		geNORM	1.426	1.483	1.426	HEL/PTB	-	UBC
Vernalisation treatment	Non-vernalised	RefFinder	3.000	1.000	1.682	UBC	HEL	PTB
		Comparative Delta Ct	1.890	1.585	1.597	UBC	HEL	PTB
		BestKeeper	1.682	1.306	1.317	UBC	HEL	PTB
		NormFinder	1.655	0.890	0.937	UBC	HEL	PTB
		geNORM	1.691	1.292	1.292	UBC/HEL	-	PTB
	Vernalised	RefFinder	1.189	3.000	1.414	PTB	HEL	UBC
		Comparative Delta Ct	1.097	1.192	1.188	PTB	HEL	UBC
		BestKeeper	1.294	1.316	1.152	HEL	PTB	UBC
		NormFinder	0.617	0.912	0.903	PTB	HEL	UBC
		geNORM	1.093	1.159	1.093	HEL/PTB	-	UBC
Developmental stage	Vegetative	RefFinder	3.000	1.414	1.189	HEL	UBC	PTB
		Comparative Delta Ct	1.521	1.392	1.204	HEL	UBC	PTB
		BestKeeper	1.309	0.438	0.986	UBC	HEL	PTB
		NormFinder	1.331	1.073	0.063	HEL	UBC	PTB
		geNORM	1.372	1.075	1.075	UBC/HEL	-	PTB
	Early reproductive	RefFinder	3.000	1.000	1.682	UBC	HEL	PTB
		Comparative Delta Ct	1.543	1.333	1.378	UBC	HEL	PTB
		BestKeeper	1.567	1.312	1.343	UBC	HEL	PTB
		NormFinder	1.304	0.736	0.907	UBC	HEL	PTB
		geNORM	1.418	1.168	1.168	UBC/HEL	-	PTB
	Late reproductive	RefFinder	3.000	1.189	1.414	UBC	HEL	PTB
		Comparative Delta Ct	1.637	1.523	1.550	UBC	HEL	PTB
		BestKeeper	1.478	1.360	1.294	HEL	UBC	PTB
		NormFinder	1.285	0.971	1.058	UBC	HEL	PTB
		geNORM	1.570	1.436	1.436	UBC/HEL	-	PTB

In all circumstances, *UBC* and *HEL* were identified by NormFinder as the most stable pair of reference genes, with an average stability value of 0.081. Using both *UBC* and *HEL*, the stability values decreased (and hence strengthened) by 0.028 and 0.045 relative to the stability value of only *UBC* when considering organ type and developmental stage, respectively. However, it was interesting to note that the use of this pair did not always result in greatest stability when considering only single factors. For example, when considering vernalisation only, the use of *UBC* as a singular reference gene provided a stability value of 0.057, whilst *UBC* and *HEL* as a pair resulted in a 0.061 stability value (*i*.*e*., slightly less stable). Similarly, the stability value when considering parental line was greater by 0.04, and therefore slightly less stable, when using *UBC* and *HEL* as opposed to using only *UBC*.

The geNorm algorithm incorporated into RefFinder largely agreed with the results from NormFinder in terms of the best pair of candidate reference genes. Overall, *UBC* and *HEL* had the least pairwise variation according to geNorm, and consequently were equally ranked as the most stable single and thus pair of candidate genes (Table 7). This pair was also observed as the most stable pair of candidate reference genes for eight of the 14 levels within factors, including: the vegetative, early reproductive and late reproductive developmental stages; the non-vernalised vernalisation treatment; the 83A:476 parental line; and the leaf, SAM and flower organ types.

## Discussion

The selection of reliable reference genes is fundamental for accurate and precise normalisation of qRT-PCR data, and consequently, for researchers to draw meaningful biological conclusions regarding patterns of gene expression determined by qRT-PCR. To our knowledge, this study provides the first validation of candidate reference genes suitable for narrow-leafed lupin, a legume grain crop of great significance to Australian agriculture. With thorough preliminary testing of seven potentially useful housekeeping genes and the use of NormFinder and RefFinder algorithms, we identified the following candidates (listed in descending order of stability) as the most reliable reference genes for this species: *UBC*, *HEL* and *PTB*.

Notably, the ranking of these three reference genes differed markedly between narrow-leafed lupin (this study) and the closely related model legume, *M*. *truncatula* [28]. In narrow-leafed lupin, the most stable reference genes were *UBC*, *HEL* and *PTB*, listed in descending order of stability. In contrast, *PTB* was the most stable gene in *M*. *truncatula*, followed by *UBC* and *HEL*, respectively. These differing rankings highlight the necessity to assess suitable candidate reference genes for each individual species, and not to assume that reference genes that are optimal in one species are optimal even in a closely related species. Furthermore, it is interesting to note that geNorm-based stability values reported by Kakar *et al*. for *PTB*, *UBC* and *HEL* were also substantially lower than those achieved for narrow-leafed lupin, adding further support to the gathering consensus in the scientific literature that the expression of housekeeping genes is by no means consistent among plant or animal species [11–18].

Similarly to Artico *et al*. [13] who validated reference genes for cotton (*Gossypium hisutum*), our study highlights the importance of rigorously assessing reference genes across a wide range of organ types representing different functions and plant developmental stages. Organ type is associated with the most significant variation in relative expression within narrow-leafed lupin and, as a result, requires a different number of reference genes for optimal normalisation. Whilst we found that *UBC* is suitable to be used as a sole reference gene when considering parental line or vernalisation treatment, optimal normalisation with respect to organ type and developmental stage requires *HEL* to be used in combination with *UBC*. Given that most organs have a unique transcriptome [43] whose composition evolves over time, it is perhaps not surprising that organ type and developmental stage are associated with greater variability in reference gene expression here. Nevertheless, this outcome suggests that the inclusion of a wide variety of organ types in future validations of any species would ensure genes with the most stable global and temporal expression possible can be established. In particular, we strongly advise that roots and reproductive organs (including flowers and pods/seed) be incorporated in future studies, as the greatest differences in relative expression of *UBC*, *HEL* and *PTB* in narrow-leafed lupin occur between these organ types.

The choice of statistical approach(es) to assess expression stability should be of careful consideration when conducting candidate reference gene assessments. We found the NormFinder algorithm to be particularly suitable for our study owing to its robust design and ability to assess gene stability according to inter- and intra-group variation in gene expression [1,13,29]. This capacity is highly advantageous when assessing a wide range of factors and numerous levels within those factors and made NormFinder our preferred algorithm. We used RefFinder in our study to test a commonly reported problem within the literature: the overall ranking of candidate reference genes can change from program to program [*e*.*g*. 1,13,42], owing to the differences among statistical algorithms [44]. RefFinder is a user-friendly platform enabling the rapid, simultaneous use of four of the most popular and widely used statistical approaches: Comparative Delta-Ct, BestKeeper, NormFinder, and geNorm. We found this software program to be a valuable and convenient method to assess differences in the perceived stability of the candidate reference genes across programs and affirm our rankings determined by NormFinder.

An important criterion for our study was the ability for the primer pairs of each candidate reference gene to discriminate between cDNA and gDNA. Whilst this characteristic is not strictly necessary for qRT-PCR experiments where gDNA is eliminated by DNase treatment prior to the synthesis of cDNA from total RNA input, it is nevertheless a useful characteristic in the event of occasional failure to prevent gDNA contamination and indeed may altogether eliminate the requirement for DNase treatment of RNA. This is of course provided that the primers for the genes of interest also span intron-exon boundaries and will not amplify other closely related isoforms, should they exist. A case in point is the observation of very high C_T_ values for No-RT controls for *PPR* and *TUB* primer pairs in this study. These values would suggest that the primers target conserved regions in a large number of related genes or among more distantly related genes that all encode a highly conserved functional domain. At this point in time, we are not able to assess sequence variability within individual members of gene families in narrow-leafed lupin due to the lack of a fully annotated genome. However, in *Arabidopsis thaliana*, there are at least 4 alpha- and 7 beta-tubulin genes [45,46], and at least 441 PPR genes [47]. In the case of the lupin *PPR*, it is therefore highly likely that the primers target multiple copies within the narrow-leafed lupin genome resulting in very high background amplification in No-RT controls.

The use of qRT-PCR primers that can reliably distinguish between DNA species is a particular advantage for researchers with large numbers of samples as it reduces financial costs and time spent on additional procedures prior to cDNA synthesis [3]. Further, avoiding exposure of RNA to chemicals associated with DNase treatment ensures the efficiency of cDNA synthesis is not compromised. For example, the activity of SuperScript®II Reverse Transcriptase has been shown to reduce by approximately 50% when cDNA synthesis reactions contain 1mM EDTA (a compound that may be used to prevent RNA degradation during heat inactivation of DNase following treatment prior to cDNA synthesis [48]) or 35% (v/v) glycerol (a component of DNase storage buffers) [49]. Thus, with the limited sequence information available to us, we have been able to identify and recommend reference genes with cost-, time- and performance-effective qRT-PCR primers.

This study represents a first effort to validate reference genes for narrow-leafed lupin. With the use of current and emerging transcriptome profiling technologies, it is likely that many more suitable reference genes will in future be identified for this species. Furthermore, such technologies will afford the opportunity for novel genes with less variable expression than traditional housekeeping genes to be discovered. An early example of this was in *Arabidopsis thaliana* where 18 new superior reference genes were identified using a large-scale public microarray data set representing more than 23,500 genes [50]. More recently, nine novel reference genes that outperform traditional housekeeping genes in terms of stability were identified in maize (*Zea mays* L.) by querying RNAseq and microarray databases [51]. Such discoveries are of great significance and should enable greater accuracy of normalisation, particularly across diverse plant organs and in other experimental conditions where traditional housekeeping genes display variability in expression.

A technology of particular promise for narrow-leafed lupin in the search for alternative reference genes is next generation sequencing, RNAseq. Unlike other methods for transcriptome-wide identification of expressed genes, such as microarrays or expressed tag sequences (ESTs), RNAseq does not rely upon prior knowledge of genomic sequences [51]. This feature is extremely advantageous for species like narrow-leafed lupin that do not yet have a published, fully annotated reference genome sequence. Kamphuis *et al*. [34] recently reported the completion of an extensive unigene assembly for cv. Tanjil, incorporating root, stem, leaf, flower and seed organ transcriptomes, using RNAseq. In addition, smaller transcriptome libraries facilitated by RNAseq have also been created for three other narrow-leafed lupin references (Unicrop, 83A:476 and P27255) [34], and four other lupin species [22,52,53]. Therefore, with valuable data sets already established, the discovery and validation of new housekeeping and novel reference genes for narrow-leafed lupin may be imminent.

## Conclusions

Quantitative reverse transcription PCR is currently one of the most popular and effective technologies available for quantifying gene expression. The successful application of this technology relies heavily upon the use of appropriate reference genes, whose expression is consistent across a number of experimental conditions. In a first attempt to validate reference genes for narrow-leafed lupin, a grain legume species, we have identified *UBC*, *HEL* and *PTB* (listed in descending order of stability) as suitable reference genes for future studies incorporating wide varieties of organ types, developmental stages, accessions and vernalisation treatments. The expression of *UBC* is sufficiently stable that it may be used as a sole reference gene for this species under the same experimental conditions as tested here. However, where resources permit, the combined use of *UBC* and *HEL* will enable optimal normalisation in studies where organ type and/or developmental stage are particularly prominent factors. In future, the use of emerging tools and the completion of valuable data sets, such as RNAseq facilitated transcriptome libraries, will make it possible for novel genes to be identified and potentially validated as valuable reference genes for narrow-leafed lupin.

## Supporting Information

S1 Table Identification of transcripts in the narrow-leafed lupin Tanjil unigene assembly of Kamphuis *et al*. [34] using *Medicago truncatula* transcripts as queries. (XLSX)

S2 Table Raw C_T_, PCR efficiency and R_2_ data for seven candidate reference genes across various organs of F_8_ RIL narrow-leafed lupins^a^. (XLSX)

S3 Table Raw C_T_ data for three promising candidate reference genes trialled across multiple organs (representing three plant developmental stages), both with and without vernalisation, in the narrow-leafed lupin F8 RIL parents, 83A:476 and P27255. (XLSX)

S4 Table Summary of p-values achieved in an Unbalanced ANOVA comparing mean CT values for three reference genes (*PTB*, *UBC* and *HEL*) in narrow-leafed lupin across organ type (cotyledon, stem, root, flower, pod, leaf, and shoot apical meristem), parental line (83A:476 and P27255) and vernalisation treatment (vernalised and non-vernalised). (PDF)

S5 Table Summary of p-values achieved in an Unbalanced ANOVA comparing mean CT values for three variables (parental line, vernalisation treatment, and plant developmental stage) in seven individual narrow-leafed lupin organ types. (PDF)

S6 Table Summary of p-values achieved in an Unbalanced ANOVA comparing mean CT values for three reference genes (*PTB*, *UBC* and *HEL*) in narrow-leafed lupin across organ type (leaves and shoot apical meristems), parental line (83A:476 and P27255), vernalisation treatment (vernalised and non-vernalised), and plant developmental stage (vegetative vs early reproductive vs late reproductive). (PDF)
