# Supplementary material for: Identifying Stable Reference Genes for qRT-PCR Normalisation in Gene Expression Studies of Narrow-Leafed Lupin (Lupinus angustifolius L.)
Source: PLoS One. 2016 Feb 12;11(2):e0148300. doi: 10.1371/journal.pone.0148300 (PMC4752343; doi:10.1371/journal.pone.0148300)
Supplement: S4 Table — (PDF) [file pone.0148300.s004.pdf]

S4 Table. Summary of p-values achieved in an Unbalanced ANOVA comparing mean C_T values for three reference genes (*PTB*, *UBC* and *HEL*) in narrow-leafed lupin across organ type (cotyledon, stem, root, flower, pod, leaf, and shoot apical meristem), parental line (83A:476 and P27255) and vernalisation treatment (vernalised and non-vernalised).

	p-value		
	PTB	***UBC***	***HEL***
Organ type	< 0.001*	<0.001*	<0.001*
Parental line	0.003*	0.003*	0.001*
Vernalisation treatment	0.286	0.068	0.008*
Organ type x[a] Parental line	0.126	0.125	0.002*
Organ type x Vernalisation treatment	0.346	0.081	0.022*
Parental line x Vernalisation treatment	0.540	0.086	0.576
Organ type x Parental line x Vernalisation treatment	0.149	0.001*	0.006*

[a] ‘x’ denotes an interaction term

* denotes a significant p-value with 95% confidence interval
