# Supplementary material for: Identifying Stable Reference Genes for qRT-PCR Normalisation in Gene Expression Studies of Narrow-Leafed Lupin (Lupinus angustifolius L.)
Source: PLoS One. 2016 Feb 12;11(2):e0148300. doi: 10.1371/journal.pone.0148300 (PMC4752343; doi:10.1371/journal.pone.0148300)
Supplement: S5 Table — (PDF) [file pone.0148300.s005.pdf]

S5 Table. Summary of p-values achieved in an Unbalanced ANOVA comparing mean C_T values for three variables (parental line, vernalisation treatment, and plant developmental stage) in seven individual narrow-leafed lupin organ types.

**Organ**	**Variable(s)**	**p-value**		
		PTB	***UBC***	***HEL***
Cotyledons	Parental line	0.768	0.714	0.841
	Vernalisation treatment	0.486	0.444	0.862
	Parental line x[b] Vernalisation Treatment	0.223	0.028*	0.098
Stems	Parental line	0.025*	0.018*	0.001*
	Vernalisation treatment	0.219	0.099	0.049*
	Parental line x Vernalisation Treatment	0.103	0.014*	0.017*
Roots	Parental line	0.088	0.028*	0.138
	Vernalisation treatment	0.126	0.039*	0.613
	Parental line x Vernalisation Treatment	0.051	0.010*	0.411
Flowers	Parental line	0.471	0.715	0.283
	Vernalisation treatment	0.744	0.901	0.073
	Parental line x Vernalisation Treatment	0.485	0.602	0.993
Pods	Parental line	0.037*	0.049*	0.009
	Vernalisation treatment	0.007*	0.073	0.006
	Parental line x Vernalisation Treatment	0.043*	0.06	0.242
Leaves	Parental line	0.069	0.031*	0.797
	Vernalisation treatment	0.869	0.012*	0.062
	Developmental stage	0.446	0.013*	0.013*
	Parental line x Vernalisation treatment	0.633	0.287	0.949
	Parental line x Developmental stage	0.012*	<0.001*	0.016
	Vernalisation treatment x Developmental stage	0.189	0.175	0.735
SAM[a]	Parental line	0.026*	0.781	0.551
	Vernalisation treatment	0.995	1.000	0.986
	Developmental stage	0.756	0.954	0.811
	Parental line x Vernalisation treatment	0.703	0.065	0.021*
	Parental line x Developmental stage	1.000	0.035*	0.075
	Vernalisation treatment x Developmental stage	0.038*	0.649	0.131

[a] Shoot apical meristem (SAM)

[b] ‘x’ denotes an interaction term

* denotes a significant p-value with 95% confidence interval
