# Supplementary material for: Identifying Stable Reference Genes for qRT-PCR Normalisation in Gene Expression Studies of Narrow-Leafed Lupin (Lupinus angustifolius L.)
Source: PLoS One. 2016 Feb 12;11(2):e0148300. doi: 10.1371/journal.pone.0148300 (PMC4752343; doi:10.1371/journal.pone.0148300)
Supplement: S6 Table — (PDF) [file pone.0148300.s006.pdf]

S6 Table. Summary of p-values achieved in an Unbalanced ANOVA comparing mean C_T values for three reference genes (*PTB*, *UBC* and *HEL*) in narrow-leafed lupin across organ type (leaves and shoot apical meristems), parental line (83A:476 and P27255), vernalisation treatment (vernalised and non-vernalised), and plant developmental stage (vegetative vs early reproductive vs late reproductive).

	p-value		
	PTB	***UBC***	***HEL***
Organ type	0.001*	0.639	<0.001*
Parental line	0.007*	0.199	0.636
Vernalisation treatment	0.583	0.182	0.082
Developmental stage	0.057	0.088	<0.001*
Organ type x[1] Parental line	0.066	0.919	0.906
Organ type x Vernalisation treatment	0.366	0.248	0.101
Organ type x Developmental stage	0.455	0.477	0.702
Parental line x Vernalisation treatment	0.814	0.048*	0.091
Parental line x Developmental stage	0.048*	<0.001*	0.002*
Vernalisation treatment x Developmental stage	0.110	0.581	0.702
Organ type x Parental line x Vernalisation treatment	0.500	0.474	0.125
Organ type x Parental line x Developmental stage	0.101	0.773	0.238
Organ type x Vernalisation treatment x Developmental stage	0.138	0.087	0.080
Parental line x Vernalisation treatment x Developmental stage	0.212	0.449	0.357

[1] 'x' denotes an interaction term

* denotes a significant p-value with 95% confidence interval
